# Genomewide binding of transcription factor Snail1 in triple‐negative breast cancer cells

**DOI:** 10.1002/1878-0261.12317

**Published:** 2018-05-21

**Authors:** Varun Maturi, Anita Morén, Stefan Enroth, Carl‐Henrik Heldin, Aristidis Moustakas

**Affiliations:** ^1^ Department of Medical Biochemistry and Microbiology Science for Life Laboratory Ludwig Institute for Cancer Research Uppsala University Sweden; ^2^ Department of Immunology, Genetics and Pathology Science for Life Laboratory Uppsala University Sweden

**Keywords:** bone morphogenetic protein, breast cancer, chromatin immunoprecipitation, epithelial–mesenchymal transition, transforming growth factor β

## Abstract

Transcriptional regulation mediated by the zinc finger protein Snail1 controls early embryogenesis. By binding to the epithelial tumor suppressor *CDH1* gene, Snail1 initiates the epithelial–mesenchymal transition (EMT). The EMT generates stem‐like cells and promotes invasiveness during cancer progression. Accordingly, *Snail1* mRNA and protein is abundantly expressed in triple‐negative breast cancers with enhanced metastatic potential and phenotypic signs of the EMT. Such high endogenous Snail1 protein levels permit quantitative chromatin immunoprecipitation‐sequencing (ChIP‐seq) analysis. Snail1 associated with 185 genes at *cis* regulatory regions in the Hs578T triple‐negative breast cancer cell model. These genes include morphogenetic regulators and signaling components that control polarized differentiation. Using the CRISPR/Cas9 system in Hs578T cells, a double deletion of 10 bp each was engineered into the first exon and into the second exon–intron junction of *Snail1*, suppressing Snail1 expression and causing misregulation of several hundred genes. Specific attention to regulators of chromatin organization provides a possible link to new phenotypes uncovered by the *Snail1* loss‐of‐function mutation. On the other hand, genetic inactivation of Snail1 was not sufficient to establish a full epithelial transition to these tumor cells. Thus, Snail1 contributes to the malignant phenotype of breast cancer cells via diverse new mechanisms.

**Abbreviations**
BMP: bone morphogenetic protein
ChIP: chromatin immunoprecipitation
CPED1: cadherin‐like and PC‐esterase domain containing 1
CRB1: Crumbs 1
ECM: extracellular matrix
EMT: epithelial–mesenchymal transition
EMT‐TF: EMT transcription factor
MET: mesenchymal–epithelial transition
PPFIA1: protein phosphatase regulatory factor interacting protein α 1
TGFβ: transforming growth factor β
ZEB: zinc finger E‐box binding homeobox 1

## 1 Introduction

During cancer progression, metastasis is a process that contributes to the mortality of cancer patients (Lambert *et al*., 2017; Nguyen *et al*., 2009). A hallmark of metastasis is the ability of tumor cells to invade their microenvironment (Lambert *et al*., 2017; Nguyen *et al*., 2009). Associated with the invasion cascade are processes of cell differentiation and de‐differentiation, which in the case of epithelial carcinomas are best known as epithelial–mesenchymal transition (EMT) and mesenchymal–epithelial transition (MET) (Nieto *et al*., 2016; Ye and Weinberg, 2015). As such differentiation changes can be reversible or often take place via successive and intermediate stages, they are also frequently described with the term epithelial plasticity (Nieto *et al*., 2016; Ye and Weinberg, 2015). EMT is driven by developmental transcription factors (EMT‐TFs) that control the expression of several genes, often classified into two broad functional groups: the epithelial genes, whose expression is repressed by the EMT‐TFs, and the mesenchymal genes, whose expression is induced by the EMT‐TFs (Nieto *et al*., 2016; Ye and Weinberg, 2015). A common net result observed at the cellular level after an EMT is the loss of cell–cell contacts and the remodeling of extracellular matrix (ECM) and cytoskeletal and cell membrane–ECM adhesion assemblies (Nieto *et al*., 2016).

A prototype EMT‐TF is Snail1/SNAI1, a member of the Snail/Scratch family of zinc finger (ZF) transcription factors, which together with Snail2/SNAI2/Slug play important roles in mediating the EMT during embryonic development and cancer progression (Barrallo‐Gimeno and Nieto, 2009; Baulida and Garcia de Herreros, 2015). Snail1 expression in epithelial cells induces the EMT (Batlle *et al*., 2000; Cano *et al*., 2000). In breast cancer, high Snail1 expression correlates with high‐grade malignancy, characterized by reduced degree of differentiation, a higher level of invasiveness and metastatic potential (Blanco *et al*., 2002). Accordingly, engineered expression of low‐level Snail1 in mice causes malignancy, suggesting that the action of Snail1 takes place at early stages of cancer progression (Perez‐Mancera *et al*., 2005). Conversely, genetic silencing of Snail1 expression in carcinomas causes MET and suppresses invasiveness (Olmeda *et al*., 2007). Snail1 can also be expressed in mesenchymal cells, such as cancer‐associated fibroblasts (CAFs), and thus drive the plasticity of the adjacent carcinoma cells (Baulida and Garcia de Herreros, 2015). In colon cancer CAFs, Snail1 controls the expression of mesenchymal cytoskeletal proteins and secretion of chemokines that coordinately promote the invasiveness of the tumor cells (Herrera *et al*., 2014). In a similar mechanistic scenario, Snail1 expression and induction of EMT also contributes to organ fibrosis as is the case of renal disease; Snail1 expression promotes renal EMT and generation of myofibroblasts with contractile features similar to those of CAFs, whereas suppression of Snail1 reverts the fibrotic phenotype in experimental mice where Snail1 expression can be switched on and off (Grande *et al*., 2015).

Snail1 is a well‐characterized transcriptional repressor, which upon direct binding to E‐box sequences (5′‐CANNTG‐3′) on gene regulatory elements, such as the promoter of the *E‐cadherin/CDH1* gene, blocks expression of E‐cadherin, a key epithelial cell–cell contact protein, thus mediating in part the detachment between differentiated epithelial cells, a hallmark of the EMT (Batlle *et al*., 2000; Cano *et al*., 2000). Upon binding to DNA, Snail1 forms complexes with corepressor complexes containing the histone deacetylases HDAC1/2, the histone H3 methyltransferases G9a and Suv39H1 and DNA methyltransferases, all enforcing transcriptional shut off, repressive chromatin conformation and even DNA methylation at the target gene, such as *CDH1*, during breast cancer EMT (Dong *et al*., 2012, 2013a; Peinado *et al*., 2004). In addition to *CDH1*, Snail1 can transcriptionally repress other epithelial genes, including the ECM gene *MUC1*, the tight junctional gene *CLAUDIN1* and the epithelial polarity gene *CRUMBS3* (Guaita *et al*., 2002; Martinez‐Estrada *et al*., 2006; Whiteman *et al*., 2008). Furthermore, Snail1 arrests the epithelial cell cycle and promotes survival, by repressing the expression of pro‐apoptotic genes (Vega *et al*., 2004), whereas transcriptional repression of the *fructose‐1,6‐bisphosphatase* (*FBP1*) gene switches on glycolytic rates and contributes to the survival of breast cancer cells that undergo the EMT (Dong *et al*., 2013b), thus linking EMT to the generation of stem‐like features in cancer cells (Ye and Weinberg, 2015). Snail1 positively regulates the expression of other EMT‐TFs, such as the ZF and homeobox transcription factor ZEB1, whose transcription is induced by a complex between Snail1 and Twist1 during breast cancer EMT in response to transforming growth factor β (TGFβ) (Dave *et al*., 2011; Guaita *et al*., 2002).

The transcriptional activity of Snail1 can be regulated by post‐translational modifications, including phosphorylation. Glycogen synthase kinase‐3β (GSK‐3β) controls Snail1 levels via three distinct mechanisms: By inactivating the transcription factor NF‐κB, GSK‐3β inhibits *Snail1* gene transcription (Bachelder *et al*., 2005); by phosphorylating Snail1 directly, GSK‐3β causes nuclear export of Snail1 (Zhou *et al*., 2004); and by phosphorylating a separate amino acid residue in Snail1, GSK‐3β recruits the ubiquitination machinery that causes degradation of cytoplasmic Snail1 (Zhou *et al*., 2004). Accordingly, the activity of GSK‐3β on Snail1 in the nucleus is counterbalanced by the small C‐terminal phosphatase that dephosphorylates and stabilizes nuclear Snail1, thus enhancing its transcriptional activity (Wu *et al*., 2009). Simultaneously, phosphorylation of nuclear Snail1 by the Lats2 protein kinase inhibits the nuclear export of Snail1 and prolongs Snail1 protein stability and transcriptional activity during the EMT (Zhang *et al*., 2012). Nuclear Snail1 also becomes poly‐ADP‐ribosylated by interacting with the chromatin‐bound enzyme poly‐ADP‐ribose polymerase 1 (PARP‐1), which also prolongs the stability and nuclear residence of Snail1, thus contributing to breast cancer EMT and invasiveness (Rodriguez *et al*., 2011). Beyond post‐translational enzymatic modifications, Snail1 protein expression is regulated at the translational level by the action of *microRNA‐34* (*miR‐34*) (Siemens *et al*., 2011). *MiR‐34* represses Snail1 protein synthesis, and *miR‐34* expression is induced by the pro‐epithelial tumor suppressor protein p53, whereas Snail1 itself represses *miR‐34* expression, thus enforcing a shutdown of its own repressor (Siemens *et al*., 2011).

Whereas *miR‐34* downregulates Snail1 expression, the best‐studied transcriptional inducer of Snail1 expression, and of EMT, in a variety of carcinomas is the TGFβ signaling pathway (Barrallo‐Gimeno and Nieto, 2009; Moustakas and Heldin, 2012). This pathway is mediated by the plasma membrane receptors of TGFβ, being serine/threonine kinases, exhibiting weak tyrosine kinase activity; these receptors phosphorylate cytoplasmic Smad proteins and other adaptor proteins that control the activity of lipid and protein kinases, coordinately leading to the regulation of target genes, such as *Snail1* (Moustakas and Heldin, 2012). In this respect, TGFβ signaling promotes the EMT, favors carcinoma invasiveness, arrests the proliferation of immune cells, and induces pro‐angiogenic factors, thus collectively enhancing metastatic potential (Bierie and Moses, 2006). Snail1 thus becomes a pivotal mediator of TGFβ actions in cancer and also controls the expression of TGFβ ligands. The mechanism by which TGFβ induces Snail1 transcription during EMT involves protein kinase signaling and Smad complexes with high mobility group A2 (HMGA2), c‐Myc, or STAT3, the latter being activated by oncogenic Ras signaling that cooperates with TGFβ during EMT induction (Peinado *et al*., 2003; Saitoh *et al*., 2016; Smith *et al*., 2009; Thuault *et al*., 2008). Furthermore, when Snail1 acts on target genes, such as *CDH1*, it cooperates with transcriptional complexes between Smads and β‐catenin/LEF‐1 (Medici *et al*., 2006; Vincent *et al*., 2009). Snail1 also feeds back to TGFβ signaling, as it can maintain the expression of the type II receptor of TGFβ in breast cancer and is required for the responsiveness of mesenchymal stem cells to TGFβ (Batlle *et al*., 2013; Dhasarathy *et al*., 2011).

Despite our deep understanding of Snail1 function and its contribution to the EMT and cancer progression, current knowledge regarding target genes of Snail remains somewhat limited. Specific key examples have been summarized above. For this reason, we analyzed the genomewide association of Snail1 in breast carcinomas and evaluated phenotypic adaptations to the loss of function mutation in Snail1 in the same tumor cells.

## 2 Material and methods

### 2.1 Cell culture

Breast cancer Hs578T and MDA‐MB‐231 cells and human embryonic kidney HEK‐293T cells were cultured in Dulbecco's modified Eagle's medium (DMEM) and breast cancer T47D cells in Roswell Park Memorial Institute (RPMI)‐1640 supplemented with 10% fetal bovine serum in the presence of penicillin and streptomycin. The cells were starved for 18 h in serum‐free DMEM or RPMI, followed by stimulation with TGFβ1 (5 ng/ml).

### 2.2 CRISPR/Cas9 knockout models

Hs578T cells were transfected with CRISPR/Cas9 and HDR plasmids targeting *Snail1*, obtained from Santa Cruz Biotech Inc. (Santa Cruz, CA, USA). Two days post‐transfection, cells were selected with puromycin and single cell colonies were selected and cultured. Knockout clones were then validated using immunoblotting and the mutated DNA sequences were analyzed using conventional PCR and DNA sequencing.

### 2.3 Transient transfection

HEK‐293T cells were seeded at a density of 5 × 10^5^ cells in 100‐mm culture dishes, and transient overexpression was performed with the indicated plasmids (final total DNA of 5 μg). Cells were transfected at approximately 80% confluency using Fugene HD (Promega Corp., Stockholm, Sweden) as transfection reagent, according to the manufacturer's guidelines. Transfection was performed for 48 h. The plasmids used were pCDNA3 as mock control and pcDNA3‐HA‐Snail1, which we have previously described (Vincent *et al*., 2009).

### 2.4 T‐Scratch assay

Cells were seeded in a six‐well plate such that they were 90% confluent the following day. A simple ‘+’ scratch was made in the cell layer using a filtered 10‐μL pipet tip. Cells were washed with PBS twice and left in DMEM. The scratched area was then observed, and pictures were obtained using a phase contrast microscope. The culture was left at 37 °C overnight and then observed under the same microscope and photographed. Both observations on day 1 and day 2 were analyzed using the t‐scratch software https://github.com/cselab/TScratch to quantify the migration of the cells. Each experiment was performed three times, and each condition included triplicates.

### 2.5 Immunoblotting and antibodies

Total cellular proteins were extracted in nonidet P‐40 (NP‐40)‐containing lysis buffer, 20 mm Tris/HCl, pH 8.0, 1% NP‐40, 150 mm NaCl, 2 mm EDTA, and complete protease inhibitor cocktail (Roche Diagnostics Scandinavia AB, Bromma, Sweden). Lysates were heated at 95 °C for 5 min and subjected to SDS/PAGE. The following antibodies were used: rabbit polyclonal anti‐Slug antibody (Santa Cruz Biotech Inc.); rabbit monoclonal anti‐Snail1 antibody (Cell Signaling, Leiden, the Netherlands); rabbit polyclonal anti‐ZEB1 antibody (Novus Biologicals, R&D Systems Europe, Abingdon, UK); rabbit control IgG‐ChIP grade, mouse control IgG‐ChIP grade and rabbit monoclonal anti‐BMP6 antibody (Abcam, Cambridge, UK); monoclonal mouse anti‐β‐actin antibody (Santa Cruz Biotech. Inc.); mouse polyclonal anti‐N‐cadherin (Becton Dickinson AB, Stockholm, Sweden); rabbit monoclonal anti‐fibronectin (Sigma‐Aldrich AB, Stockholm, Sweden); rabbit polyclonal anti‐coxsackie and adenovirus receptor (anti‐CAR; a gift of Jonas Fuxe, Karolinska Institute, Sweden); rabbit polyclonal anti‐HSP95 (home‐made); and rabbit polyclonal anti‐CPED1 antibody (Thermo Fisher Scientific, Stockholm, Sweden).

### 2.6 DNA affinity precipitation (DNAP)

HEK‐293T cells transfected with pcDNA3‐HA‐Snail1 were lysed in 0.5% NP‐40, 100 mm EDTA, and 100 mm Tris/HCl, pH 8.0. Lysates were precleared with streptavidin beads for 30 min and incubated for 90 min with biotin‐labeled double‐stranded oligonucleotides. Streptavidin beads were then added for 30 min followed by three washes with lysis buffer and dissolved into sample buffer containing 1% SDS and 1 mm DTT. DNA‐bound proteins were subjected to SDS/PAGE followed by immunoblotting. The DNA sequences represented in the biotinylated oligonucleotides were obtained from the Snail1‐binding motif information as described in the Section 4; they represent sequences centering on peaks defined by the ChIP analysis and map in each corresponding human gene body as shown in the figures. The oligonucleotide sequences were as follows: *hsCPED1*, forward, 5′‐biotin‐GTCCGCAAATGCACATCAGGCTTCACCAGCTAATGAGGACAAATGAGGTC‐3′; reverse, 5′‐GACCTCATTTGTCCTCATTAGCTGGTGAAGCCTGATGTGCATTTGCGGAC‐3. *HsBMP6*, forward, 5′‐biotin‐GATACCACTTGGCCAATCCATGACAAGGTCCATGAACAAATGGCCTTG‐3′; reverse, 5′‐CAA GGCCATTTGTTCATGGACCTTGTCATGGATTG GCCAAGTGGTATC‐3′. *HsCRB1*, forward, 5′‐biotin‐GACCCACCAGATGACTCTGGGCACACAGAATAATTG‐3′; reverse, 5′‐CAATTATTCTGTGTGCCCAGAGTCATCTGGTGGGTC‐3′. *HsPPF1A1*, forward, 5′‐biotin‐CTATATTCTGTTGTTGGGTGGTGTGTTCTATG‐3′; reverse, 5′‐CATAGAACACACCACCCAACAACAGAATATAG‐3′. *HsPPF1A*mut, forward, 5′‐biotin‐CTATATTCTATTATTAGGTAGTATATTTCTATG‐3′; reverse, 5′‐CATAGAAATATACTACCTAATAATAGAATATAG‐3′. *HsCDH1*‐A, forward, 5′‐biotin‐GGGGCTCACCTGGCTGCAG CCAC‐3′; reverse, 5′‐GTGGCTGCAGCCAGGTGAGCCCC‐3′. *HsCDH1*‐B, forward, 5′‐biotin‐GGCCGGCAGGTGAACCCTCAGCC‐3′; reverse, 5′‐GGCTGAGGGTTCACCTGCCGGCC‐3′.

### 2.7 Chromatin immunoprecipitation

Cells were cultured and then fixed in 2% formaldehyde for 10 min at 37 °C. Immediately, formaldehyde was removed and cells were washed in ice‐cold PBS twice. Cells were scraped in PBS and spun down at 4000 rpm for 5 min. The supernatant was removed, and cells were lysed in SDS lysis buffer (1% SDS, 10 mm EDTA, 50 mm Tris, pH 8.1, with added protease inhibitors) for 20 min on ice. Lysed cells were then sonicated to obtain an average DNA fragment size of 250 bp. Input chromatin of about 10% was aliquoted and frozen at −20 °C. The remaining lysate was then diluted 10 times in ChIP dilution buffer (0.01% SDS, 1.0% Triton X‐100, 1.2 mm EDTA, 16.7 mm Tris/HCl pH 8.1, 167 mm NaCl, supplemented with protease inhibitors) and subjected to immunoprecipitation using the Snail1 antibody described above or control rabbit antiserum, overnight at 4 °C. Protein‐A dynabeads were added to the lysate, and incubation was prolonged for 2 h at 4 °C. Beads were then washed once in low salt buffer (0.1% SDS, 1% Triton X‐100, 2 mm EDTA, 20 mm Tris/HCl, pH 8.1, 150 mm NaCl), once in high salt buffer (0.1% SDS, 1% Triton X‐100, 2 mm EDTA, 20 mm Tris/HCl pH 8.1, 500 mm NaCl), once in lithium chloride wash buffer (0.25 M LiCl, 1% IGEPAL, 1% deoxycholic acid, 1 mm EDTA, 10 mm Tris/HCl, pH 8.1), and then twice in TE buffer (10 mm Tris/HCl, pH 8.0, 1 mm EDTA). After the series of washing steps, beads and input samples were resuspended in elution buffer (1% SDS, 0.1% of 1 m NaHCO_3_) and mixed up and down for 30 min and left for de‐crosslinking in the presence of NaCl at 65 °C overnight. The chromatin was then subjected to proteinase‐K digestion followed by phenol–chloroform extraction. Respective input was used to normalize the DNA in each sample that was subjected to chromatin immunoprecipitation. The extracted DNA was then subjected to either PCR quantification or sequencing experiment. ChIP‐seq results were validated using ChIP‐qPCR with specific primers for the human *CDH1* promoter, forward 5′‐GGCCCTGCAGTTCCTTGGCT‐3′, reverse 5′‐AGTGAGCAGCGCAGAGGCTG‐3′; human *BMP6* promoter, forward 5′‐GCTCTCACTTGGGGTTCACTA‐3′, reverse 5′‐CAC CCAATGGAACTTCAAGGC‐3′; human *PPFIA1*, forward 5′‐TGTCTGTGATGGTCCTGTAGG‐3′, reverse 5′‐ CTGTACAGGACCCCACT‐3′; human *CRB1*, forward 5′‐CCTGACCTCGTGATCCAACT‐3′, reverse 5′‐GTCAAGAATGTGCACTCCTCA‐3′; human *CPED1*, forward 5′‐ TTAGAGGCCAGATAACCTGCAC‐3′, reverse 5′‐GAGGTTTCCAACCTTGCCGA‐3′.

### 2.8 DNA library preparation and sequencing protocols

ChIP DNA was obtained with four biological and technical replicates and pooled for sequencing. The quality of the ChIP DNA obtained was analyzed with a Bioanalyzer. The DNA obtained from input, and ChIP was sheared with sonication using the Covaris S2 instrument (Covaris, Inc., Brighton, UK). ChIP‐seq libraries were constructed from the sheared samples using the AB Library Builder System (Life Technologies, Stockholm, Sweden), followed by amplification and wildfire conversion according to the manufacturer's protocols. Sequencing was performed at 75 bp read length on the SOLiD 5500W system (Life Technologies). Library preparation was performed using the library kit (5500 SOLiD Library Builder Fragment Core Kit + 5500W Conversion Primer Kit), after which sequencing was performed using a sequencing instrument (SOLiD 5500W) at sequencing unit SOLiD5500W FlowChip. Raw sequences were aligned to the human genome hg19 using maximum stringency, and unique sequences were retained in the .BAM file format. Raw sequences were aligned to the human genome hg19 using Life Technologies/Thermo Fisher lifescope (version 2.1) (Stockholm, Sweden) with default settings retaining only uniquely mapped reads.

### 2.9 Real‐time RT‐PCR

RNA was extracted from both Hs578T wild‐type and *Snail1* knockout clones using the TRIzol reagent protocol (Ambion, Life Technologies). Complementary DNA (cDNA) was synthesized using the iScript cDNA synthesis kit from Bio‐Rad (Bio‐Rad Laboratories AB, Nacka, Sweden). Real‐time PCR was carried out using iTaq SYBR green supermix with ROX from Kappa (Techtum, Nacka, Sweden) using denaturation temperature 95 °C for 30 s, annealing temperature 56 °C for 30 s, and amplification temperature 72 °C for 45 s, repeating this protocol 39 times; a melting curve was plotted using 0.5 °C raise for every 5 s from 65 °C to 95 °C. The primers used for quantitative PCR amplification were as follows: human *HPRT1* forward 5′‐ GCTTCCTCCTCCTGAGCAGTC‐3′ and reverse 5′‐CACTAATCACGACGCCAGGGCTGC‐3′; human *PPFIA1* forward 5′‐GGTGTTCACGGAGCACTTCT‐3′ and reverse 5′‐CCTTCTATCAGTCCCCATGACCAA‐3′; *CRB1* forward 5′‐GCCTCTGATCCGTGTG TCA‐3′ and reverse 5′‐ACTGAGCCAATAGTGGTGAAAATGT‐3′; *BMP6* forward 5′‐GGACATGGTCATGAGCTTTGTGAA‐3′ and reverse 5′‐CAGTCCTTGTAGATGCGGAATTCT‐3′; and *CPED1* forward 5′‐CCCCACAACTGCCAATATGGT‐3′ and reverse 5′‐CTGCCATTCCTGCAACGTTT‐3′.

### 2.10 AmpliSeq transcriptome human gene expression

RNA for AmpliSeq was extracted with three biological replicates and three technical replicates. Total RNA (50 ng) was reverse‐transcribed to cDNA using Ion AmpliSeq™Transcriptome Human Gene Expression Kit Preparation Protocol (Revision A.0; Life Technologies). The acquired cDNA was amplified using Ion AmpliSeq™ Transcriptome Human Gene Expression core panel (Life Technologies), and the primer sequences were then partially digested. Then, adaptors (Ion P1 Adapter and Ion Xpress™ Barcode Adapter, Life Technologies) were ligated to the amplicons. Adaptor‐ligated amplicons were purified using Agencourt^®^ AMPure^®^ XP reagent (Beckman Coulter AB, Bromma, Sweden) and eluted in amplification mix (Platinum^®^ PCR SuperMix High Fidelity and Library Amplification Primer Mix, Life Technologies) and amplified. Size selection and purification were conducted using Agencourt^®^ AMPure^®^ XP reagent (Beckman Coulter AB). The amplicons were quantified using the Fragment Analyzer™ instrument (Advanced Analytical Technologies, Inc., Ankeny, IA, USA) with DNF‐474 High Sensitivity NGS Fragment Analysis Kit (Advanced Analytical Technologies, Inc.). Samples were then pooled (six or less per pool), followed by emulsion PCR on either the Ion OneTouch™ 2 System using the Ion PI™ Hi‐Q™ OT2 Kit (Life Technologies), or on the Ion Chef™ System using the Ion PI Hi‐Q Chef Kit (Life Technologies). The pooled samples were loaded on Ion PI™ v3 chips and sequenced on the Ion Proton™ System using the Ion PI™ Hi‐Q Sequencing 200 Kit chemistry (200 bp read length, Life Technologies). Acquired reads were aligned to the hg19 AmpliSeq Transcriptome ERCC v1 using the Torrent Mapping and Alignment Program (tmap) with default settings. Differentially expressed genes were called requiring a mean log_2_‐fold change over the replicates of two and a *q*‐value < 0.05 (one‐sided *t*‐test with false discovery rate adjustment).

### 2.11 Bioinformatic analysis methods

#### 2.11.1 Transcriptomic analysis in the GOBO database

The gene expression data sets for *SNAI1* and *SNAI2* in human breast cancer cells were obtained by utilizing the cell line module of the Web‐based tool gene expression‐based Outcome for Breast cancer Online (GOBO) (Ringner *et al*., 2011). Data were obtained using default settings and protocols prescribed by the authors.

#### 2.11.2 ChIP‐seq analysis

Aligned reads were filtered on mapping quality using samtools (Li *et al*., 2009) with ‘–q 20’. Peaks were called from ChIPs with the input as background using macs software (version 1.4.2) (Zhang *et al*., 2008) with the following changes to default settings: ‘–nomodel –shiftsize=125 –keep‐dup=1’. Identified peaks were annotated to the closest gene using BEDTools (Quinlan and Hall, 2010) and protein coding genes from the refseq databases (hg19). FASTA sequence (Human Genome version GRCh37.57) ± 125 bp from the center of each peak summit as predicted by MACS was extracted, and enriched DNA motifs were identified using TOMTOM motif identification suit (Bailey *et al*., 2009). Data were obtained using default settings and prescribed protocols by the authors.

#### 2.11.3 Visualization of peaks

ChIP‐seq peaks were uploaded into the UCSC genome browser (Kent *et al*., 2002) using custom bigWig tracks and overlayed using publicly available H3K27AC data.

#### 2.11.4 Gene ontology

Differentially expressed genes and binding targets of Snail1 were classified into the groups of molecular, biological, and cellular components using the gene ontology (GO) Panther online tool (Mi *et al*., 2013). Genes were grouped into functional groups using default settings and protocols.

## 3 Data accessibility

All ChIP‐seq and AmpliSeq transcriptomic data have been deposited to Array Express under Accession Numbers E‐MTAB‐5242 and E‐MTAB‐5244, respectively.

## 4 Results

### 4.1 Mesenchymal Hs578T breast cancer cells express high levels of Snail1

**Figure 1:**
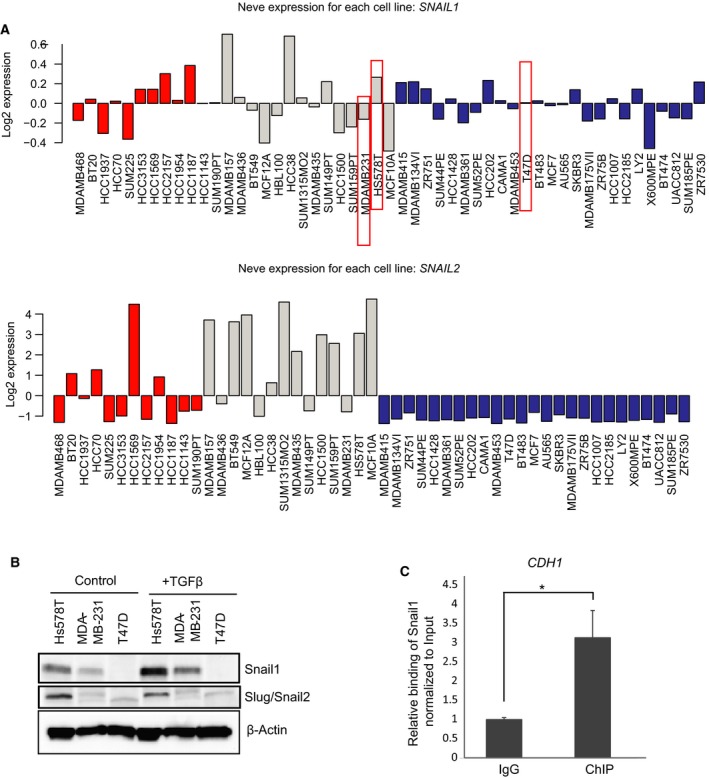
Hs578T breast cancer cells express high Snail1 levels. (A) Expression of *SNAIL1* and *Slug/SNAIL2* mRNA in different subtypes of breast cancers cell lines (basal‐A, red; basal‐B, gray; and luminal, blue) based on expression values derived from the GOBO database. (B) Immunoblot analysis showing protein expression of Snail1, Slug, and loading control β‐actin in Hs578T, MDA‐MB‐231, and T47D cells stimulated with vehicle (control) or TGFβ (5 ng·mL^−1^) for 9 h. (C) ChIP‐qPCR showing the significant enrichment (0.1% of the total input) of the *CDH1* promoter region in a ChIP experiment using the Snail1 antibody, relative to the enrichment by nonspecific IgG. Statistical significance *P‐value = 0.037 is shown based on a Student's *t*‐test where *n* = 3, and average values along with SD are shown.

Snail1 can be highly expressed in human breast cancers, which correlates with the grade of malignancy, de‐differentiation, and metastatic dissemination (Blanco *et al*., 2002). Using an independent breast cancer mRNA expression database, GOBO (Ringner *et al*., 2011), we confirmed that *Snail1* mRNA expression scored highly in diverse human breast cancer cell lines, including basal‐B (highest expression), some basal‐A (intermediate expression), and fewer luminal epithelial (lowest expression) breast cancer cells (Fig. 1A). Confirming variability in cancer patient expression profiles, not all basal‐B cells exhibited robust mRNA levels for *Snail1* (Fig. 1A). We also examined expression of the sibling EMT‐TF, *Snail2* in the same cohort of breast cancer cells, and also observed high *Snail2* mRNA expression in the basal‐B subgroup of human breast cancer cells (Fig. 1A). Similar data can be obtained from transcriptomic analyses of breast cancer tissue (data not shown); however, in such whole tumor tissue databases, it is not clear whether the specific expression profile represents the carcinoma cells or various stromal cells in the tumor microenvironment. For this reason, we limited our database analysis to isolated carcinoma cell lines that have been cultured *in vitro* and, based on their clonality, are known to be free of associated tumor stromal cells. Overall, the strong level of expression and a significant correlation between *Snail1* and *Snail2* expression in human breast cancer cells of the basal‐B subgroup are consistent with previous findings, and agree with the EMT‐like phenotype of basal‐B breast cancer cells (Hennessy *et al*., 2009; Shipitsin *et al*., 2007; Taube *et al*., 2010).

Although mRNA expression profiles are widely used, they do not always correlate with corresponding protein expression. For this reason, we screened two triple‐negative breast cancer cells of the basal‐B subgroup (Hs578T and MDA‐MB‐231) and one cell model of luminal epithelial breast cancer (T47D) for expression of Snail1 protein (Fig. 1B). This was necessitated by our aim to perform quantitative chromatin immunoprecipitation‐sequencing (ChIP‐seq) analysis in the tumor cells. We found that Hs578T cells of the basal‐B subgroup expressed high or detectable levels of endogenous Snail1 protein (Fig. 1B), in agreement with the mRNA analysis (Fig. 1A). The Snail1 protein levels of Hs578T cells were higher than the levels expressed in another mesenchymal and highly aggressive basal‐B breast cancer cell model, MDA‐MB‐231 cells (Fig. 1B). We also stimulated the cells with TGFβ1 to further enhance expression of Snail1 protein and observed significant induction of Snail1 in both Hs578T and MDA‐MB‐231 cells (Fig. 1B). Contrary to what was expected based on the mRNA profile (Fig. 1A), T47D luminal epithelial breast cancer cells did not express detectable Snail1 protein levels (Fig. 1B). Snail1 protein expression also correlated with Snail2/Slug protein levels (Fig. 1B).

Based on the above analysis, we selected Hs578T cells as the cell model for ChIP‐seq analysis. As the best‐established target of Snail1 transcriptional repression activity is *E‐cadherin*/*CDH1*, we confirmed that the endogenous Snail1 protein of these cells (Fig. 1B) could be detected bound to the *CDH1* promoter, using ChIP‐qPCR analysis (Fig. 1C). Endogenous Snail1 was readily detectable as bound to the *CDH1* promoter of Hs578T cells when compared to the input and unspecific IgG antibody. This assay also served as validation for the utility of Hs578T cells for the ChIP‐seq experiment.

### 4.2 A few hundred genomic sites are recognized by Snail1 in triple‐negative breast cancer cells

**Figure 2:**
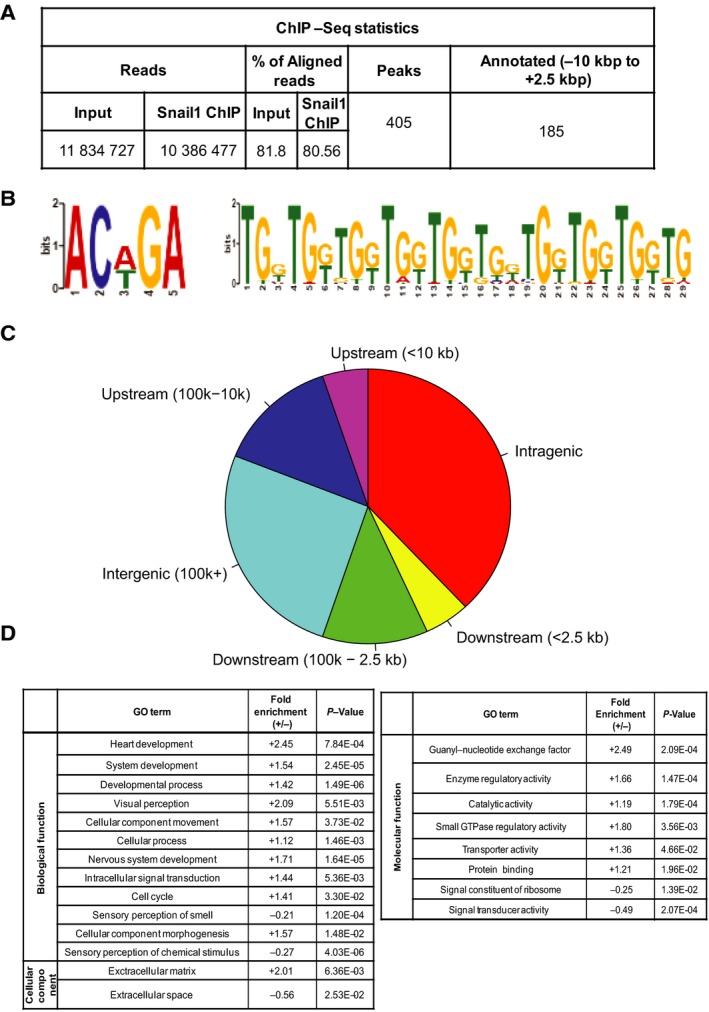
Snail1 associates with hundreds of genomic sites in triple‐negative breast cancer cells. (A) Table showing data and flow of analysis of a Snail1 ChIP‐seq experiment from sequence reads to annotated gene assignment of the Snail1 binding sites. (B) Visualization of statistically significant DNA motifs, derived from the Snail1 binding site sequences in Hs578T cells using the TOMTOM motif comparison tool. The sequence on the left is not, whereas the sequence to the right is centrally enriched. Sequence position is graphed on the *x*‐axis versus probability of frequency (bits) on the *y*‐axis. (C) Pie chart showing the relative location of Snail1 binding peaks on the Hg19 genome with respect to annotated genes. (D) GO analysis of annotated genes from the Snail1 ChIP‐seq peaks into molecular function, biological process and cellular component categories using the GO Panther database. Relative fold enrichment and *P*‐values indicate significance of each functional category.

High‐yield ChIP using the Hs578T cells and the Snail1 antibody characterized in Fig. 1B, C was followed by sequence analysis (ChIP‐seq; Fig. 2A). Almost 405 sequence peaks were detected (after normalization relative to input chromatin), with a rate of alignment of the sequenced reads of roughly 80% in both input and immunoprecipitated chromatin DNA samples (Fig. 2A, Table S1). All of these peaks were annotated to 185 genes defined based on sequences relative to the transcriptional start site (TSS), 10 kbp upstream to 2.5 kbp downstream of various genes (Fig. 2A).

We then identified the top DNA sequence motifs recognized by Snail1, revealing a TAL1/GATA1 sequence motif (ACA/TGA) and a repetitive TGG motif that is known to be recognized by the transcription factors RREB1, RUNX2, and PAX4 (Fig. 2B). The classical E‐box motif (CANNTG) to which Snail1 has been previously shown to bind (Barrallo‐Gimeno and Nieto, 2009; Baulida and Garcia de Herreros, 2015) was also identified but with lower statistical confidence (data not shown and Section 4 below). The repeated TGG motif was centrally enriched, whereas the ACA/TGA motif was not, suggesting possible direct regulation of target genes that encompass the TGG motif by Snail1. Further analysis of the 405 peaks revealed that their majority belonged to intragenic regions (Fig. 2C). The remaining peaks corresponded to other genomic regions extending from −100 to +100 kbp relative to the gene TSS (Fig. 2C).

Gene ontology enrichment analysis revealed that Snail1 associates with genes of diverse functional categories, including embryonic and central nervous system development, sensory perception, and heart development (Fig. 2D). Snail1 target genes showed a high fold of enrichment (+2.49) in the class of guanyl nucleotide exchange factors that participate in various signaling pathways (Fig. 2D). On the super class of cellular components, GO analysis showed that Snail1‐targeted genes had a higher relevance and fold enrichment toward the class of ECM genes (Fig. 2D).

### 4.3 Novel gene targets of Snail1 in breast cancer cells

**Figure 3:**
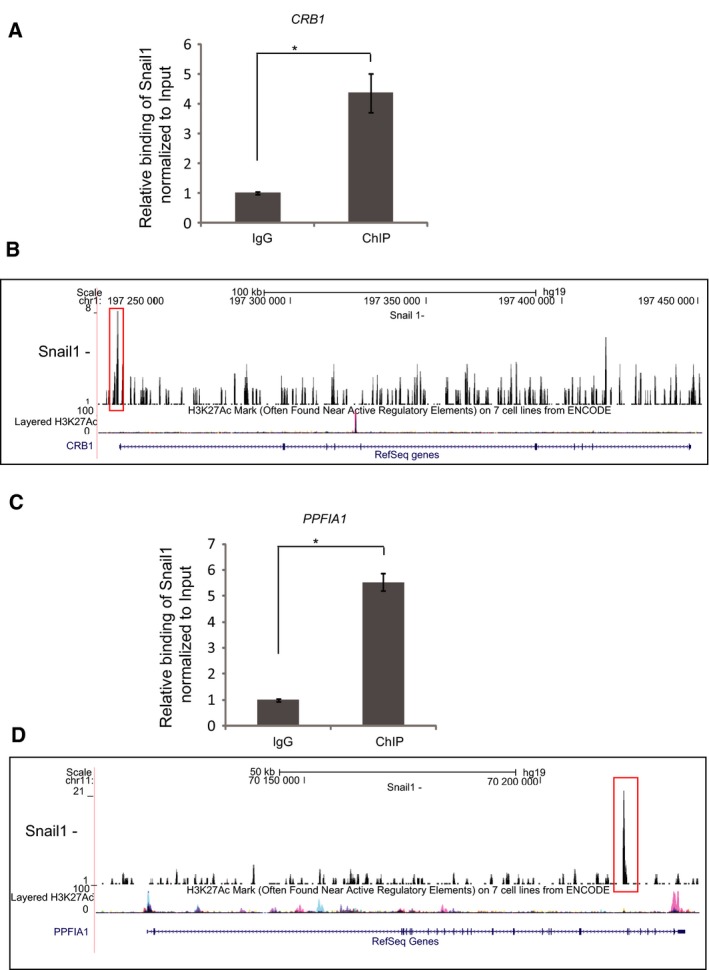
Novel gene targets of Snail1 in breast cancer cells. (A, C) ChIP‐qPCR showing the significant enrichment of the *CRB1* (0.02% of the total input) (A) and *PPFIA1* (0.05% of the total input) (C) promoter regions in a ChIP experiment using the Snail1 antibody, relative to the enrichment by nonspecific IgG. Statistical significance *P‐value = 8.76E‐04 and *P‐*value = 2.37E‐05, respectively, is shown based on a Student's *t*‐test where *n *= 3, and average values along with SD are shown. (B, D) Representation of Snail1 binding to the *CRB1* (B) and *PPFIA1* (D) genes; ChIP‐seq peaks (marked in red box) were aligned with tracks of H3K27Ac ChIP‐seq, which is used as a marker of gene activity, based on data available on the database, using the UCSC genome browser.

Among the functional categories of genes to which significant association of Snail1 was measured (Fig. 2D), we validated the *Crumbs1* (*CRB1*) gene that belongs to the cell polarity group and the *protein phosphatase regulatory factor interacting protein* α (*PPFIA1*) that belongs to the liprin family of regulators of the interaction between adhesion receptors and the ECM (Fig. 3). ChIP‐qPCR analysis based on primers corresponding to the DNA peaks identified in the ChIP‐seq experiment revealed that Snail1 binds to *CRB1* and *PPFIA1* significantly when compared to the input and unspecific IgG antibody (Fig. 3A, C). We then used the UCSC genome browser that provides location information of the pattern of several histone modifications, such as histone H3 lysine 27 acetylation (H3K27Ac), which mark transcriptional enhancer regions. We found that Snail1 binds near genomic regions that exhibit high transcriptional activity in these two genes (Fig. 3B, D). Nine additional genes were also validated for Snail1 binding using ChIP‐qPCR (data not shown).

**Figure 4:**
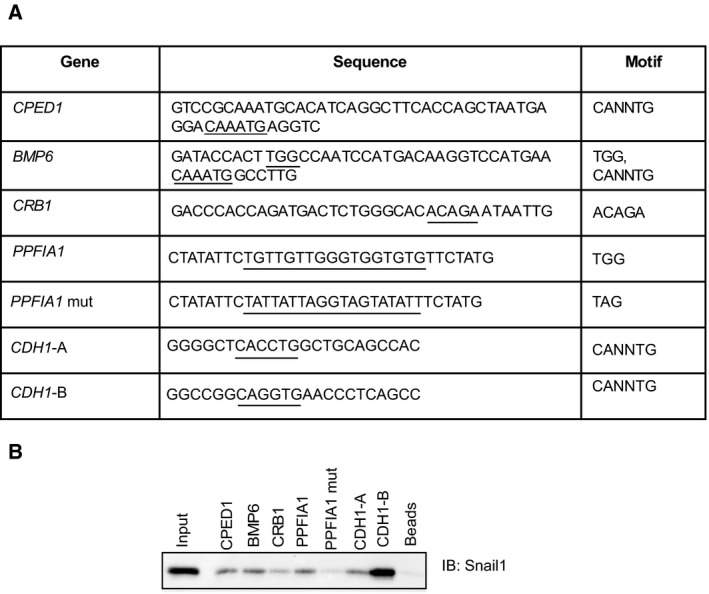
Binding of Snail1 to newly identified DNA sequences. (A) Selected genes and DNA sequences from these genes where Snail1 binding peaks were identified by ChIP‐seq (which were synthesized as biotinylated oligonucleotides), and corresponding motifs included in each gene sequence. (B) Binding of Snail1 protein expressed in transfected HEK‐293T cells and analyzed by DNAP using the specific DNA oligonucleotides of panel A. Input shows total cell lysate from the same cells prior to application to the biotinylated oligonucleotides and beads show negative control of streptavidin beads plus cell lysate in the absence of DNA.

As ChIP assays do not prove direct binding of a transcription factor to a specific DNA sequence, but rather binding of the transcription factor to a chromatin locus to which a specific DNA sequence is closely associated, we performed DNA affinity precipitation (DNAP) experiments using synthetic double‐stranded oligonucleotides (Fig. 4). We selected four novel genes identified in the ChIP‐seq experiment and *CDH1* as positive control (Fig. 4A). The five selected genes represent the three major DNA binding motif classes, the classical E‐box CANNTG (*CPED1*,* BMP6* and *CDH1*), the TGG motif (*BMP6* and *PPF1A1*), and the ACAGA motif (*CRB1*) (Fig. 4A); the oligonucleotide sequences were derived based on the center of specific peaks from the ChIP‐seq experiment. The bone morphogenetic protein 6 (BMP6) sequence included adjacent TGG and E‐box elements (Fig. 4A). To further increase the specificity of the assay, we employed DNAP experiments using total cell lysates from HEK‐293T cells transiently transfected with a plasmid expressing human Snail1 protein (Fig. 4B). Although this approach does not test purified, recombinant Snail1 protein, it provides highly expressed cellular Snail1, which acquires potentially necessary modifications that regulate its DNA binding activity. Figure 4B clearly demonstrates that all four (*CPED1*,* BMP6*,* CRB1*, and *PPF1A1*) newly identified Snail1‐binding sequences bound significant levels of cellular Snail1, to a comparable degree as one of the two E‐box motifs of the *CDH1* promoter (*CDH1‐A*, mapping at −35 to −13 bp relative to the TSS). The second *CDH1* E‐box motif (*CDH1‐B*, mapping at −86 to −64 bp relative to the TSS) bound Snail1 with much higher affinity (Fig. 4B). As specificity and negative controls, we used streptavidin beads that gave very low background binding and a mutant form of the *PPFIA1* sequence (*PPFIA1‐mu*t), which also resulted in low background binding, confirming the importance of the TGG motif (Fig. 4B). Based on the above experiments, we conclude that the genomewide analysis of Snail1 binding can be reproduced by ChIP‐qPCR and DNAP assays.

### 4.4 Snail1 knockout reduces the ability of Hs578T cells to migrate

**Figure 5:**
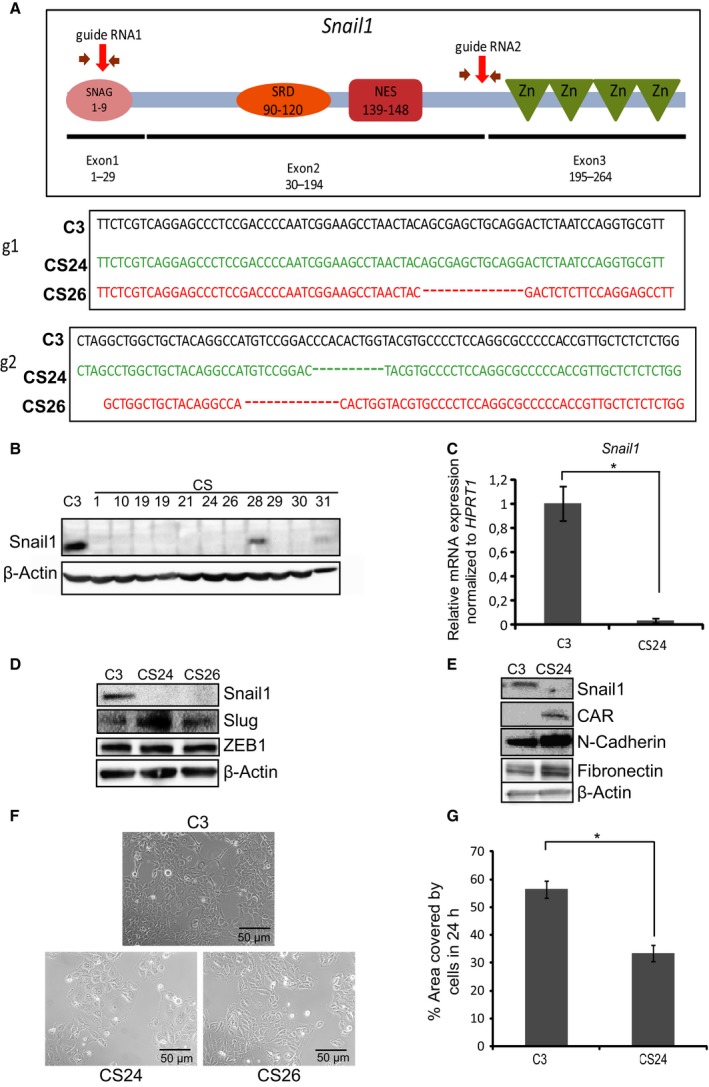
*Snail1* knockout generates an intermediate phenotype and suppresses cell migration of Hs578T cells. (A) DNA sequences of the *Snail1* gene in control C3 cells and deleted nucleotides in *Snail1* knockout clones CS24 and CS26 after CRISPR‐/Cas9‐mediated knockout using specific gRNA‐containing plasmids (red arrows on the *Snail1* gene cartoon). The latter graphs the human Snail1 protein with its functional domains, SNAG regulatory domain, serine‐rich domain (SRD), nuclear export signal (NES), and ZF, along with the corresponding three exons and numbering of the amino acid residues. (B) Immunoblot analysis showing Snail1 protein levels along with β‐actin levels that serve as protein loading control, in Hs578T cell clones C3 (control), Snail1 KO clones CS1, CS10, CS19, CS19 less protein, CS21, CS24, CS26, CS28, CS29, CS30, and CS31. (C) Quantification of *Snail1* mRNA expression levels in C3 and CS24 clones. Statistical significance *P‐value = 0.0003 is shown based on a Student's *t*‐test where *n* = 3, and average values along with SD are shown. (D) Immunoblot analysis showing levels of Snail1, Slug, ZEB1, and β‐actin proteins in C3, CS24, and CS26 clones. (E) Protein analysis of Snail1, CAR, N‐cadherin, fibronectin, and β‐actin, in C3 and CS24 clones. (F) Phase contrast images showing cell morphology in C3, CS24, and CS26 Hs578T cell clones. Bars represent 50 μm. (F) Quantification of wound healing assays by the T‐scratch software, showing % of wound closure area 24 h after a scratch was made in C3 and CS24 Hs578T cell clones. Statistical significance *P‐value = 0.0007 is shown based on a Student's *t‐*test where *n* = 3, and average values along with SD are shown.

To confirm the existence of genes that are directly regulated by Snail1, we generated a complete knockout of the *Snail1* gene in the Hs578T cells using the CRISPR/CAS9 gene editing protocol. We selected guideRNAs (gRNAs) that targeted two distinct regions of the *Snail1* mRNA: the middle of exon1 that encodes for the Snail1 SNAG domain, and the exon2–intron2 junction (Fig. 5A, top panel), and cotransfected vectors expressing these gRNAs simultaneously into Hs578T cells. The selected stably transfected cells exhibited deletions of 10 bp each in the exon2–intron2 junction (CS24 clone) or deletions of 10 bp in both exon1 and the exon2–intron junction (CS26), as compared to control cells (C3) that carried the wild‐type DNA sequences (Fig. 5A, bottom panel). Several individual cell clones were selected, and most of them exhibited complete loss of Snail1 protein expression with a knockout success rate of more than 50%. Of nine Snail1 knockout clones that were characterized, two clones were further analyzed in deeper detail. Immunoblot analysis confirmed undetectable levels of Snail1 protein expression in clones CS24 and CS26 and in many additional knockout clones (Fig. 5B); further expression analysis of clone CS24 also showed dramatic loss of *Snail1* mRNA expression (Fig. 5C). The knockout was specific for Snail1 and did not affect the related protein Snail2/Slug or the functionally related EMT‐TF ZEB1 (Fig. 5D). In fact, the Snail1 knockout cells exhibited slightly enhanced Slug protein levels (Fig. 5D), whereas ZEB1 levels remained constant in all three tested cell clones. Protein expression analysis of additional markers of epithelial (CAR) and mesenchymal (N‐cadherin, fibronectin) cells revealed that Snail1 knockout resulted in a relative good expression of the epithelial CAR, but also slightly stronger expression of the mesenchymal N‐cadherin and fibronectin (Fig. 5E). The latter is compatible with a phenotypic switch toward an intermediate phenotype that combines both mesenchymal and epithelial features (Nieto *et al*., 2016; Ye and Weinberg, 2015), an observation that requires deeper analysis.

Another key phenotypic aspect that distinguishes epithelial from mesenchymal cells is their morphology (Nieto *et al*., 2016); microscopic analysis of the knockout cells showed a weak but observable change in cell morphology of CS24 and CS26 cells compared to control CS3 cells (Fig. 5F), which is also compatible with the generation of a mixed mesenchymal–epithelial phenotype, an observation that requires further analysis. In a T‐scratch cell migration assay, the CS24 cells exhibited significant delay in their migration relative to the control C3 cells (Fig. 5G). Thus, the Hs578T breast cancer cells expressed some distinguishable phenotypic changes upon loss of Snail1 expression, possibly indicating the generation of an intermediate differentiation stage toward the epithelial state.

### 4.5 Analysis of gene expression in Hs578T cells carrying a *Snail1* knockout

**Figure 6:**
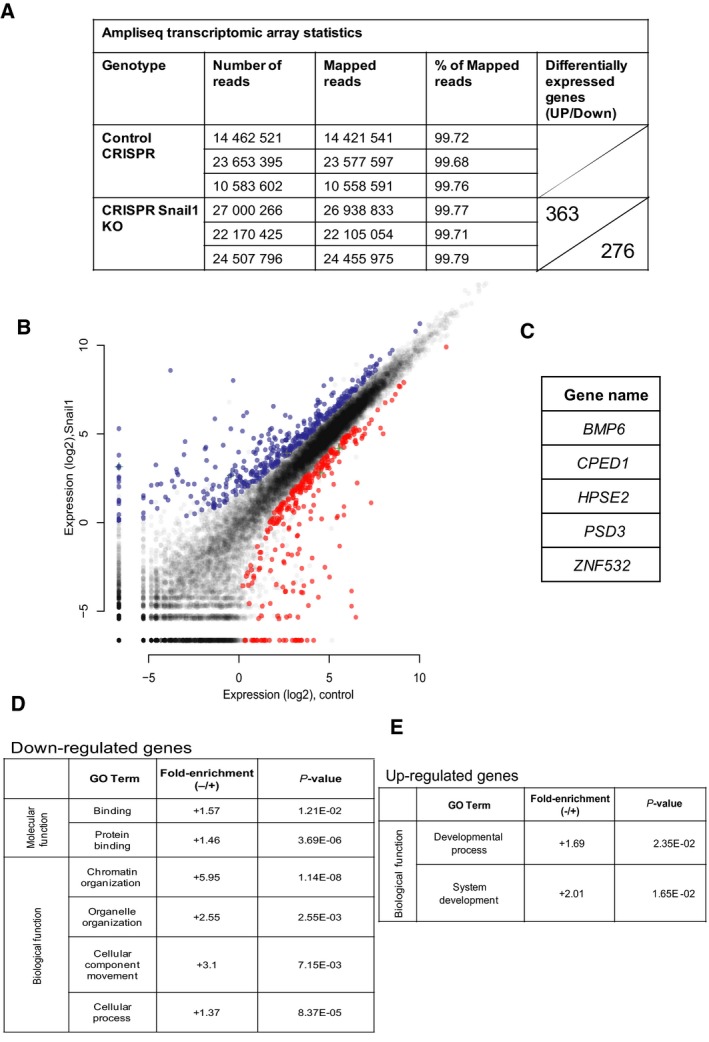
Transcriptomic analysis of genes regulated by Snail1. (A) Table showing data and flow of analysis using AmpliSeq transcriptomic arrays measuring gene expression in triplicate biological replicates of C3 (Control CRISPR) and CS24 (CRISPR *Snail1* KO) Hs578T cell clones, from number of reads obtained to number of differentially expressed genes (upregulated: top sector, and downregulated: bottom sector). (B) Profile of all the Ref‐Seq genes based on their relative expression in the CS24 Hs578T cell clone plotted against the C3 Hs578T cell clone. Upregulated genes in the *Snail1* knockout clone (blue) and downregulated genes (red color) along with statistically nonsignificant expressed genes (gray color) are plotted, and genes identified using the ChIP‐seq analysis (Fig. 1) are superimposed using a plus symbol. (C) List of the five genes that are marked with plus symbol in panel B. (D, E) GO analysis of annotated genes from the *Snail1* knockout AmpliSeq experiment, classified into molecular function, biological process and cellular component categories using the GO panther database. Relative fold enrichment and *P* values indicate significance of gene classification in each functional category. The data are divided into (D) differentially expressed and significantly downregulated genes upon *Snail1* knockout and (E) differentially expressed and significantly upregulated genes upon *Snail1* knockout.

The clean knockout system generated in the triple‐negative breast cancer Hs578T cells gave us the opportunity to perform a transcriptomic assay, using the AmpliSeq analysis that measures expression levels of all Ref‐Seq genes (Fig. 6). The assay uses targeted enrichment of over 21 000 genes, whose levels where measured in control C3 and *Snail1* knockout CS24 cells (Fig. 6). This analysis produced data for 16 to 24.5 m reads out of which, over 99% aligned to the reference human genome (Fig. 6A, Table S2). After normalization of knockout (CS24) cell expression to control (C3) Hs578T gene expression, we identified 363 upregulated (blue dots) and 276 downregulated (red dots) genes, respectively, with the majority of expressed genes showing lack of change in expression (gray dots) (Fig. 6A, B). This transcriptomic analysis cannot discriminate between direct and indirect gene targets of Snail1. Unexpectedly, a substantial number of genes were downregulated in *Snail1* knockout cells, suggesting that Snail1 positively regulates transcription, and this class of genes included primarily DNA binding, chromatin regulators, and genes involved in organelle organization and intracellular movement (Fig. 6D). On the other hand, Snail1 could repress a large set of genes involved in embryonic development (Fig. 6D). Among the genes whose expression remained constant after *Snail1* knockout were the EMT‐TF genes *Snail2/Slug*,* Twist1*,* and ZEB1* (Fig 6B, gray dots and not shown), which translated to weak Slug protein induction and stable ZEB1 protein expression (Fig. 5D).

When the ChIP‐seq and transcriptomic array data were compared, only a few specific genes scored significantly in this overlap; five genes appear as direct Snail1 targets when the AmpliSeq expression data are overlayed with ChIP‐seq results (Fig. 6B, cross symbols; Fig. 6C, Table S2). GO analysis on this small subset of genes (*BMP6*,* CPED1*,* HPSE2*,* PSD3,* and *ZNF532*) did not show significant enrichment for any functional class of genes (data not shown), because of the low number of genes analyzed. The collective data suggest that Snail1 regulates the expression of several hundreds of genes, and a small subset may be direct target genes in triple‐negative breast cancer cells.

### 4.6 Regulators of bone homeostasis under the control of Snail1 in Hs578T cells

Among the 639 genes whose expression changed significantly upon knockout of Snail1 (Fig. 6), we paid further attention to two specific regulators of bone homeostasis because triple‐negative breast cancer cells often metastasize to the human skeleton and form osteolytic lesions (Nguyen *et al*., 2009), but more importantly because these two genes were regulated in an opposite manner, and Snail1 could associate with their respective genomic regulatory sequences (Fig. 4).

**Figure 7:**
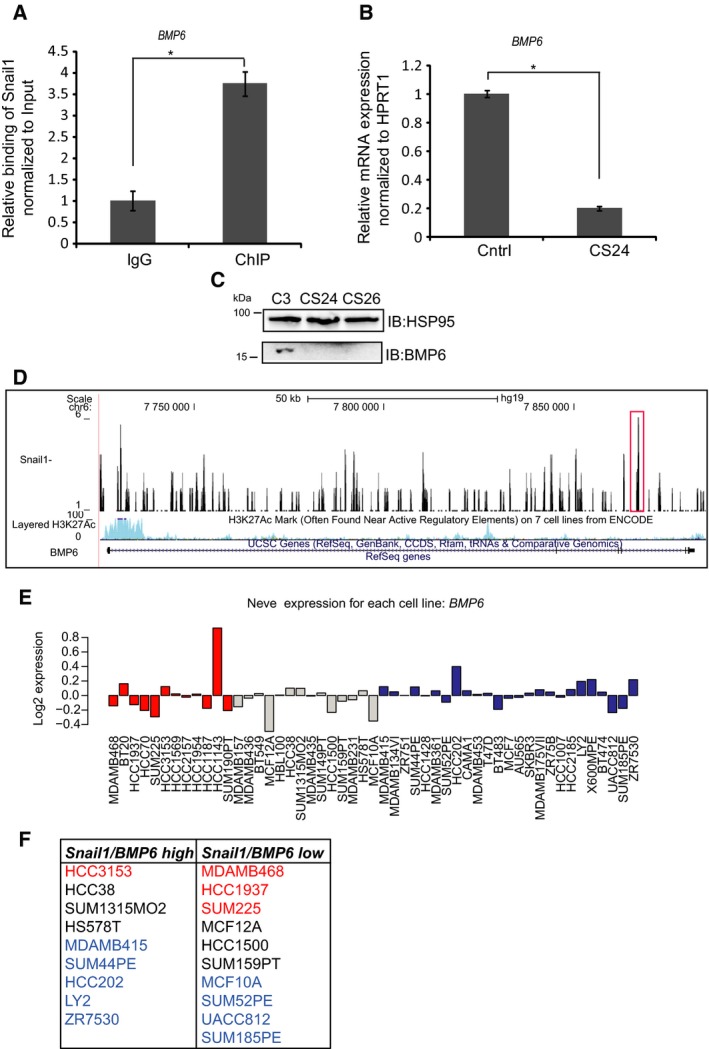
Snail1 regulates BMP6. (A) ChIP‐qPCR showing a significant enrichment (0.3% of the total input) of the *BMP6* promoter region in a ChIP experiment using the Snail1 antibody, relative to the enrichment by nonspecific IgG. Statistical significance *P‐value = 0.0002 is shown based on a Student's *t*‐test where *n *= 3, and average values along with SD are shown. (B) Measurement of relative amount of *BMP6* mRNA expressed in C3 and CS24 Hs578T cells after normalization with the *HPRT1* housekeeping mRNA. Statistical significance *P‐value = 1.32E‐06 is shown based on a Student's *t*‐test where *n *= 3, and average values along with SD are shown. (C) Protein analysis of loading control HSP95 and mature monomeric BMP6 in C3, CS24, and CS26 clones. (D) Representation of Snail1 binding to the *BMP6* gene; obtained ChIP‐seq peaks (marked in red box) and with tracks of H3K27Ac ChIP‐seq, which is used as a marker of enhancer activity, based on data available in the database, using the UCSC genome browser. (E) Expression of *BMP6* mRNA in different subtypes of breast cancer cell lines (basal‐A, red; basal‐B, gray; and luminal, blue) based on expression values derived from the GOBO database. (F) The analysis of *BMP6* mRNA expression using the GOBO database showed nine breast cancer cell lines with high *SNAIL1* and high *BMP6* expression and 10 with low *SNAIL1* and low *BMP6* expression. Each cell line is color‐coded according to their division in basal‐A, red; basal‐B, black; and luminal, blue.

Bone morphogenetic protein 6 is a member of the TGFβ family that regulates bone development and is also involved in the process of stem cell differentiation and a specific metabolic pathway of iron homeostasis in the liver (Parrow and Fleming, 2014; Vukicevic and Grgurevic, 2009). The *BMP6* gene scored both in the ChIP‐seq and transcriptomic analysis. ChIP‐qPCR quantification showed significant binding of Snail1 to the *BMP6* promoter region (Fig. 7A), as mapped by DNAP (Fig. 4B). Upon Snail1 knockout, CS24 cells clearly demonstrated a significant reduction in *BMP6* mRNA expression (Fig. 7B), which was nicely translated to the protein level as analyzed in clones CS24 and CS26 (Fig. 7C). These data suggest that the binding of Snail1 to this gene correlates with a positive regulatory activity on the expression of *BMP6* mRNA. The comparative ChIP‐seq analysis further revealed that the transcriptional enhancer marker H3K27Ac also exhibited strong enrichment along the *BMP6* gene body (Fig. 7D). To extend the data generated from the Hs578T cell model, we queried all the human breast cancer cell models included in the GOBO database (Fig. 7E) and identified 19 cell lines in which high‐level *Snail1* correlated with high *BMP6* or, inversely, low‐level *Snail1* correlated with low‐level *BMP6* (Fig. 7F). Interestingly, such positive correlations were not unique to the basal‐B group of breast cancer cells, but could be identified in basal‐A and luminal breast cancer cells, suggesting that Snail1 may regulate BMP6 expression in a large cohort of breast cancers.

**Figure 8:**
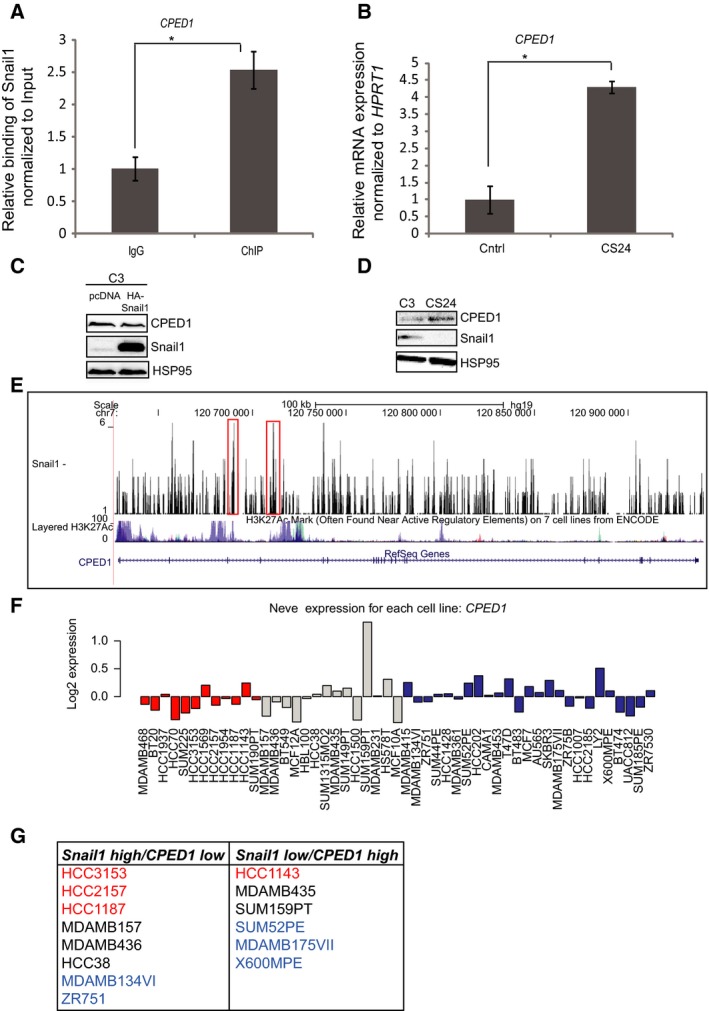
*CPED1* is a novel target gene of Snail1. (A) ChIP‐qPCR showing a significant enrichment (0.2% of the total input) of the *CPED1* promoter region in a ChIP experiment using the Snail1 antibody, relative to the enrichment by nonspecific IgG. Statistical significance *P‐value = 0.0015 is shown based on a Student's *t*‐test where *n* = 3, and average values along with SD are shown. (B) Measurement of relative amount of *CPED1* mRNA expressed in C3 and CS24 Hs578T cells after normalization with the *HPRT1* housekeeping mRNA. Statistical significance *P‐value =* *0.0002 is shown based on a Student's *t*‐test where *n* = 3, and average values along with SD are shown. (C) Protein analysis of CPED1, Snail1, and HSP95 in C3 clones transfected with pcDNA3 empty vector and HA‐Snail1 plasmid. (D) Protein analysis of CPED1, Snail1, and HSP95 in C3 and CS24 clones. (E) Representation of Snail1 binding to the *CPED1* gene; obtained ChIP‐seq peaks (marked in red box) and with tracks of H3K27Ac ChIP‐seq, which is used as a marker of enhancer activity, based on data available in the UCSC genome browser. (F) Expression of *CPED1* mRNA in different subtypes of breast cancers cell lines (basal‐A, red; basal‐B, gray; and luminal, blue) based on expression values derived from the GOBO database. (G) The analysis of *CPED1* mRNA expression using the GOBO database showed eight breast cancer cell lines with high *SNAIL1* and low *CPED1* expression and six with low *SNAIL1* and high *CPED1* expression. Each cell line is color‐coded according to their division in basal‐A, red; basal‐B, black; and luminal, blue.

The *cadherin‐like and PC‐esterase domain containing 1* (*CPED1*) gene was also occupied by Snail1 in the ChIP‐seq analysis; ChIP‐qPCR assay confirmed the specificity of binding over control immunoglobulin and after normalization to the input DNA (Fig. 8A), and DNAP confirmed Snail1 binding to a short motif of the *CPED1* gene (Fig. 4B). The expression of *CPED1* mRNA was measurable in control C3 Hs578T cells and became significantly increased after *Snail1* knockout in CS24 cells (Fig. 8B). Analyzing endogenous CPED1 protein expression in C3 cells transiently transfected with a human Snail1‐expressing vector confirmed relative downregulation of CPED1 (Fig. 8C), whereas CPED1 protein was significantly upregulated in the Snail1 knockout clone CS24 (Fig. 8D), in agreement with the mRNA profile in the same cells (Fig. 8B).

Multiple peaks of Snail1 binding associated with the *CPED1* gene, many of which overlapped with the H3K27Ac marks on the chromatin of this gene (Fig. 8E). *CPED1* is a gene that has been genetically linked via genomewide association studies to the regulation of bone mass in humans (Chesi *et al*., 2015). The molecular and cellular function of CPED1 protein remains unknown. These data suggest that Snail1 actively represses the *CPED1* gene in the triple‐negative breast cancer cells, and upon loss of Snail1, the gene becomes de‐repressed, which enhances its expression. We therefore suggest that *CPED1* may represent a gene whose transcriptional repression by Snail1 may be important for a subset of breast cancers. We queried the human breast cancer cell collection of the GOBO database (Fig. 8F) and identified 14 cell lines in which high‐level *Snail1* correlated with low *CPED1,* or inversely, low‐level *Snail1* correlated with high‐level *CPED1* (Fig. 8G). Similar to the analysis of *BMP6* expression, the data suggest that Snail1 may regulate CPED1 expression in all subtypes of breast cancers.

## 5 Discussion

Snail1 is a transcription factor, whose function is intimately linked to the process of EMT, tumor cell invasive and metastatic abilities, and the generation of stem‐like cancer cells (Barrallo‐Gimeno and Nieto, 2009; Baulida and Garcia de Herreros, 2015; Lambert *et al*., 2017). Today, we understand several details of how Snail1 mediates the EMT by repressing target genes such as *CDH1* and a few other genes involved in epithelial cell polarity and cell–cell adhesion (Baulida and Garcia de Herreros, 2015; Nieto *et al*., 2016). However, few of the EMT studies focusing on Snail1 function have been performed in human cancer cells that naturally express Snail1; they have rather been performed in relatively normal or benign tumor cells engineered to overexpress Snail1 after transfection.

Based on this reality, we studied triple‐negative breast cancer cells (Fig. 1) and analyzed the prolife of association of Snail1 at the genomewide level, which revealed several hundred genomic loci (Fig. 2). Using CRISPR/Cas9 as a tool to genetically inactivate endogenous *Snail1* in the cancer cells (Fig. 5), we identified several hundreds of genes, whose expression was affected by the absence of Snail1 (Fig. 6), suggesting that Snail1 endogenously supports a significant genetic program of the breast cancer cell. This experimental approach resulted in the identification of several previously unappreciated genes, whose expression can be regulated by Snail1, and furthermore, it functionally established that removal of a major EMT‐TF from the triple‐negative breast cancer cells showed significant repression of cell motility (Fig. 5); however, it was sufficient to induce only partial or weak signs of reversion to a more epithelial phenotype, reflecting an intermediate phenotype of cell differentiation (Fig. 5).

Previous studies associated the transcriptional repressive activity of Snail1 with the loss of expression of various epithelial genes in tumor cells, including the cell–cell contact genes *CDH1* (Batlle *et al*., 2000; Cano *et al*., 2000) and *CLAUDIN1* (Martinez‐Estrada *et al*., 2006), the ECM component *MUC1* (Guaita *et al*., 2002), and the polarity regulator *CRUMBS3* (Whiteman *et al*., 2008). In addition, Snail1 represses pro‐apoptotic genes and the *FBP1* gene, thus promoting the metabolism of cancer stem cells (Dong *et al*., 2013b; Vega *et al*., 2004).

The analysis presented here has revealed a significant number of previously not appreciated potential targets of Snail1 transcriptional activity (Figs 2, 6). This probably reflects the origin of the host cell, triple‐negative breast cancer cells Hs578T. The Hs578T cells belong to the claudin‐low subgroup of basal‐B breast cancers and exhibit strong mesenchymal features (Fig. 1A) (Hennessy *et al*., 2009; Shipitsin *et al*., 2007; Taube *et al*., 2010). It is worth noting that at the onset of this work, we attempted to analyze genomewide Snail1 occupancy in several cell models of breast cancer; however, we identified very few whose endogenous Snail1 protein level permitted quantitative ChIP‐seq (Fig. 1B). This is in contrast to a wide array of transcriptomic analyses that report high levels of *Snail1* mRNA in a variety of breast cancers, including the breast cancer cell lines analyzed in Fig. 1A. To a certain approximation, the difference between *Snail1* mRNA and protein levels can be justified based on the well‐established regulation of Snail1 stability post‐translationally, via phosphorylation, ubiquitin‐mediated degradation, or miRNAs (Díaz and de Herreros, 2016).

Based on ChIP‐Seq analysis, we have identified several hundreds of genomic sites where Snail1 binds, many of which mapped within a range of 10 kbp or less from the TSS of well‐characterized gene bodies (Fig. 2A, C). Snail1 is known to bind to the E‐box of the proximal *CDH1* (Batlle *et al*., 2000; Cano *et al*., 2000). Using the genomewide data, we could identify two additional DNA sequence motifs that represented the majority of the binding sites where Snail1 localized (Fig. 2B). These are the TAL/GATA1 motif and the repetitive TGG motif of RREB1/RUNX2/PAX4, suggesting that Snail1 exhibits a rather restrictive binding specificity when compared to other transcription factors. In our ChIP‐seq experiment, the E‐box was also identified but with lower statistical confidence (data not shown). Validation of the ChIP‐seq analysis using ChIP‐qPCR clearly demonstrated binding of Snail1 to the *CDH1* promoter that encompasses the E‐boxes in Hs578T cells (Fig. 1C) and to four representative genes among those newly identified (Figs 3A, C, 7A, 8A). Further validation by DNAP confirmed that all identified sequence motifs were genuine in terms of the ability of Snail1 to associate with them with high affinity, which is comparable to the binding affinity on the *CDH1* E‐box (Fig. 4B).

The method of genetic inactivation of Snail1 using CRISPR/Cas9‐based recombination proved rather successful (Fig. 5). A similar approach was earlier used in an ovarian cancer cell model (RMG‐1 cells) and demonstrated that knockout of Snail1 generated ovarian cancer cells with stronger cell–cell contacts and a cytoskeletal organization that resembled that of epithelial cells (Haraguchi *et al*., 2015). A similar phenotypic change was observed in the breast cancer Hs578T cells, as the cells adopted tighter cell–cell contacts and reduced migratory ability (Fig. 5F, G). However, similar to the ovarian cancer cells, Hs578T cells lacking Snail1 protein expression did not revert to fully epithelial cells. This is partly because of the redundant function of the multiple EMT‐TFs that are highly expressed in the Hs578T cells, such as Snail2/Slug and ZEB1 (Fig. 1B). The expression of these two EMT‐TFs remained strong in the *Snail1* knockout cells, and Slug was even slightly induced (Fig. 1B, gray dotes). Thus, loss of a single EMT‐TF can support a MET and promote epithelial characteristics in tumor cells, but the presence of other EMT‐TFs compensate by providing strong enough signals that support the mesenchymal differentiation of such breast cancer cells. The protein marker analysis (Fig. 5E) supports this conclusion and suggests that the Snail1 knockout Hs578T cells best resemble an intermediate phenotype that coexpresses both mesenchymal and epithelial proteins. Furthermore, the migratory ability of Hs578T cells relies significantly on the function of Snail1 as these knockout cells exhibited lower migratory ability (Fig. 5G). The lack of reversion of E‐cadherin expression after *Snail1* knockout implies an alternative mechanism for the reduced migratory phenotype of the knockout cells, which may involve other genes transcriptionally induced by active Snail1.

The transcriptomic analysis generated a list of 639 genes whose levels changed after *Snail1* knockout (Fig. 6). Many of these genes provide fresh ideas regarding the function of Snail1 in breast cancer. For example, whereas transcriptional repression of the polarity gene *CRUMBS3* by Snail1 has been firmly established (Whiteman *et al*., 2008), we now provide evidence that Snail1 binds to the related member of the Crumbs family, *CRB1* (Fig. 3A, B). CRB1 expression is highly regulated when the epithelial polarity and morphogenesis changes during carcinoma progression (Bazellières *et al*., 2009; Halaoui and McCaffrey, 2015). This is because CRB1 makes direct contact with adaptor proteins such as zonula occludens 3 and facilitates the assembly of epithelial tight junctions (Roh *et al*., 2002). Disassembly of tight junctions is a hallmark of EMT (Nieto *et al*., 2016), and thus, binding of Snail1 to the *CRB1* gene may indicate an extensive transcriptional network whereby Snail1 transcriptionally represses several members of the epithelial polarity machinery. A second gene that exhibits strong binding of Snail1 is *PPFIA1* (Fig. 3C, D). Liprin family adaptors, including Liprin‐α1/PPFIA1, regulate cell motility, invadopodia, and ECM degradation in breast cancer cells (Astro *et al*., 2011; Chiaretti and de Curtis, 2016). In addition, PPFIA1 mediates organization of the vimentin cytoskeleton (Pehkonen *et al*., 2016). Regulation of liprin expression by EMT‐TFs has not been previously appreciated. It is therefore possible that transcriptional induction of *PPFIA1* by Snail1 may contribute to the invasive phenotype of breast cancer cells and the induction of vimentin intermediate filament assembly, a hallmark of EMT. Such functional relevance for *CRB1* and *PPFIA1* gene regulation by Snail1 awaits future investigation. Snail1 also binds and positively regulates *BMP6* gene expression (Fig. 7). Whereas BMP6 is best known for its contribution to mesenchymal stem cell biology and the regulation of iron metabolism in the liver (Parrow and Fleming, 2014; Vukicevic and Grgurevic, 2009), evidence links BMP6 to the process of MET. Specifically, BMP6 signaling leads to downregulation of ZEB1 and preservation of epithelial features in breast cancer cells, whereas breast cancer cells undergoing EMT exhibit loss of BMP6 expression due to methylation of DNA sequences on the *BMP6* gene (Liu *et al*., 2014; Yang *et al*., 2007). These reports suggest that Snail1 might act as a transcriptional repressor of *BMP6*. However, our data suggest a positive role for Snail1 in *BMP6* expression (Fig. 7). We propose that the coordinate expression of ZEB1, Snail2, and Snail1 in the Hs578T cells supports *BMP6* expression, whereas loss of Snail1 in the knockout cell clones reveals a dominant role of the other EMT‐TFs in repressing BMP6, whose expression is reduced and thus possibly indirectly contributes to the sustained expression of ZEB1 (Fig. 5D). A last example is the *CPED1* gene, which is repressed by Snail1 binding and whose expression is de‐repressed upon *Snail1* knockout (Fig. 8). The biology of CPED1 remains unexplored, and so far, this gene is linked to bone disorders based on genomewide association studies (Chesi *et al*., 2015). It is therefore intriguing to propose that *CPED1* repression by Snail1 may be linked to the maintenance of BMP6 expression; however, a molecular link between BMP6 signaling and CPED1 function remains to be tested. Taken together, the gene expression analysis supports both positive and negative regulation of transcription by Snail1.

## 6 Conclusion

In conclusion, our work on Snail1 in human breast cancer cells has generated an extensive resource for novel discoveries in the context of Snail1‐mediated transcriptional regulation and EMT. This study also opens the interesting possibility of identifying Snail1 target genes, whose function may be possible to be suppressed together with the loss of Snail1 function, and thus generates breast cancer cells with more epithelial features (MET), thus providing means for synthetic lethality that could eliminate these breast cancer cells. Loss of the prosurvival signals generated by Snail1 promise such an approach in the continuous attempt to generate conditions that elicit tumor eradication.

## Author contributions

AM performed conception. VM and AM performed design. AM and VM acquired data. AM, VM, and SE analyzed data. VM, SE, CHH, and AM interpreted data. VM, SE, CHH, and AM involved in article drafting and critical revision for important intellectual content. VM, SE, CHH, and AM finally approved the study prior to publication.

## Supporting information


**Table S1.** An Excel file including primary data from the ChIP‐Seq analysis.This file tabulates in an organized manner the raw data deposited in Array Express under accession number E‐MTAB‐5242. The data are organized as: chromosome number, peak start, peak end, MACS, peak score, gene start, gene end, gene name, transcribed strand and distance from the gene start.

**Table S2.** An Excel file presenting the primary data from the AmpliSeq analysis.This file tabulates in an organized manner the raw data deposited in Array Express under accession number E‐MTAB‐5244. The data are organized as: Gene name, Target ID, ENTREZ_GENE_ID, normalized expression values in triplicate samples of the mock C3 clone (up_242_1, up_242_2, up_242_3) and the Snail1 KO CZ1 clone (up_242_7, up_242_8, up_242_9), mean of the mock C3 clone expression (meanCtrl), mean of the Snail1 KO CZ1 clone expression (mean Snail1), fold‐change in expression between Snail1 KO CS24 and control C3 cells (absMeanFC), p value (pval), adjusted p value (padj), differentially expressed gene (DEG) and coincidence with the ChIP‐Seq peaks (chipSeqPeak).
